# Genetic Determinants of Altered Virulence of Type O Foot-and-Mouth Disease Virus

**DOI:** 10.1128/JVI.01657-19

**Published:** 2020-03-17

**Authors:** Fan Yang, Zixiang Zhu, Weijun Cao, Huanan Liu, Ting Wei, Min Zheng, Keshan Zhang, Ye Jin, Jijun He, Jianhong Guo, Xiangtao Liu, Haixue Zheng

**Affiliations:** a State Key Laboratory of Veterinary Etiological Biology, National Foot and Mouth Diseases Reference Laboratory, Key Laboratory of Animal Virology of Ministry of Agriculture, Lanzhou Veterinary Research Institute, Chinese Academy of Agricultural Sciences, Lanzhou, China; University of Texas Southwestern Medical Center

**Keywords:** FMDV, S fragment, virulence, genetic determinant, host range, foot-and-mouth disease virus

## Abstract

Under different circumstances, the alteration of several viral genes may give an evolutionary advantage to the virus to maintain its prevalence in nature. In this study, a 70-nucleotide deletion in the small fragment (S fragment) of the viral 5′-untranslated region (5′-UTR) together with one amino acid insertion in the leader protein (L^pro^) that naturally occurred in several serotype O foot-and-mouth disease virus (FMDV) strains in China was identified. The properties of two field serotype O FMDV strains, with or without the 70-nucleotide deletion in the S fragment and the amino acid insertion in L^pro^, were compared *in vitro* and *in vivo*. Clinical manifestations of FMD were clearly observed in cattle and pigs infected by the virus without the mutations. However, the virus with the mentioned mutations caused FMD outcomes only in pigs, not in cattle. To determine the role of the 70-nucleotide deletion in the S fragment and the single amino acid insertion in L^pro^ in the pathogenicity and host range of FMDV, four recombinant viruses, with complete genomes and a 70-nucleotide deletion in the S fragment, a single amino acid insertion in L^pro^, or both mutations, were constructed and rescued. It showed that deletion of 70 nucleotides in the S fragment or insertion of one amino acid (leucine) at position 10 of L^pro^ partly decreased the viral pathogenicity of Mya-98 lineage virus in cattle and pigs. However, the virus with dual mutations caused clinical disease only in pigs, not in cattle. This suggested that the S fragment and L^pro^ are significantly associated with the virulence and host specificity of FMDV. The naturally occurring dual mutation in the S fragment and L^pro^ is a novel determinant of viral pathogenicity and host range for serotype O FMDV.

**IMPORTANCE** FMD is probably the most important livestock disease in the world due to the severe economic consequences caused. The alteration of several viral genes may give the virus selective advantage to maintain its prevalence in nature. Here, we identified that a 70-nucleotide deletion in the S fragment combined with a single leucine insertion in the leader protein (L^pro^) is a novel determinant of restricted growth on bovine cells, which significantly contributes to the altered virulence of serotype O FMDV in cattle. A synergistic and additive effect of the 70-nucleotide deletion in the S fragment and the single leucine insertion in L^pro^ on the virulence and host specificity of the virus was determined. These results will benefit efforts to understand the vial pathogenicity mechanism and molecular characteristics of FMDV.

## Abstract (precis)

FMD is probably the most important livestock disease in the world due to the severe economic consequences caused. The alteration of several viral genes may give the virus selective advantage to maintain its prevalence in nature. Here, we identified that a 70-nucleotide deletion in the S fragment combined with a single leucine insertion in the leader protein (L^pro^) is a novel determinant of restricted growth on bovine cells, which significantly contributes to the altered virulence of serotype O FMDV in cattle. A synergistic and additive effect of the 70-nucleotide deletion in the S fragment and the single leucine insertion in L^pro^ on the virulence and host specificity of the virus was determined. These results will benefit efforts to understand the vial pathogenicity mechanism and molecular characteristics of FMDV.

## INTRODUCTION

Foot-and-mouth disease (FMD) is a highly contagious viral disease of cloven-hoofed animals. The rapid and extensive spread of FMD often results in trade restrictions and high economic losses (1). Foot-and-mouth disease virus (FMDV) is the etiological agent of FMD, which belongs to genus *Aphthovirus*, family *Picornaviridae*. FMDV includes seven major serotypes, i.e., O, A, C, Asia 1, SAT1, SAT2, and SAT3, showing poor cross-protective activity and high genetic variability (2). Serotype O is the most common serotype worldwide and causes serious outbreaks in China. Most FMDV strains infect all susceptible host species. However, some FMDV strains have a restricted host range. For example, serotype O Cathay strains affect only pigs (3). Several PanAsia lineage strains caused only clinical disease in cattle or affected only pigs (4). Nonstructural protein (NSP) 3A has been determined to be a genetic determinant of altered host tropism of an FMDV outbreak in Taiwan in 1997 (3). A partial deletion in 3A was shown to attenuate serotype O FMDV in cattle (5).

The genome of FMDV is a single positive-sense strand of RNA of about 8.0 kb in length, which is artificially divided into the 5′ untranslated region (UTR), the open reading frame (ORF), and the 3′ UTR. The ORF sequence encodes four structural proteins (VP1, VP2, VP3, and VP4) and various nonstructural proteins (L^pro^, 2A, 2B, 2C, 3A, 3B, 3C, 3D, 3AB, and 3ABC) (1). FMDV leader protein (L^pro^) is one of the main antagonistic factors of the virus and is widely known to cleave various host proteins and suppress host antiviral activity, which contribute to virus replication (6). L^pro^ blocks host antiviral responses by means of different mechanisms, such as cleaving the host transcription factor and inhibiting alpha/beta interferon (IFN-α/β) production (6). L^pro^ has a proteinase activity, self-cleaves from the nascent viral polyprotein precursor during FMDV replication, and plays important roles in viral pathogenesis (7). Mutation of several functional domains of L^pro^ dramatically impairs the pathogenicity of FMDV for the challenged hosts (8, 9).

The 5′ UTR and 3′ UTR of FMDV are significantly involved in viral RNA replication and are required for viral replication (10). The FMDV 5′ UTR contains several distinct elements with approximately 1,300 nucleotides (nt), including the small fragment (S fragment), a poly(C) tract, several pseudoknots, a stable stem-loop structure termed the *cis*-acting replication element (*cre*), and the internal ribosome entry site (IRES) (11, 12). The first portion of the 5′ UTR (about 350 nt) is the S fragment consisting of a long stem-loop. The S fragment of poliovirus is only about 80 nt long and is involved in viral RNA replication and stability (13, 14). As for the S fragment of FMDV, previous studies have suggested that it plays a role in viral replication and affects viral pathogenesis (15). The poly(C) tract is about 150 to 200 nt in field FMDV strains and may regulate virus replication (16, 17). Our previous study indicates that pseudoknots are related to viral pathogenicity and viral host range (18). The *cre* is about 55 nt in length and is required for FMDV RNA replication (12). The IRES is about 450 nt in length and directs the initiation of protein synthesis on the viral RNA (19, 20).

An unexpected deletion of 70 nt within the S fragment of the 5′ UTR of several Mya-98 lineage strains of the Southeast Asia topotype of serotype O FMDV (O/SEA/Mya-98) isolated in China has been reported (21, 22). However, the role of this deletion in FMDV remains unknown (21). In the present study, we found that all O/SEA/Mya-98 FMDV strains with the 70-nt deletion were isolated from pigs. For all of the previously reported bovine origin O/SEA/Mya-98 strains (with 5´-UTR sequence information available in GenBank), no deletions were observed in the S fragment. Meanwhile, we found that a single amino acid insertion existed in L^pro^ of O/HKN/20/2010, which included the 70-nt deletion within the S fragment (22). This single amino acid insertion in L^pro^ was in concurrence with the 70-nt deletion in the S fragment in all of these O/SEA/Mya-98 virus strains. To determine whether this deletion in the S fragment and a single amino acid insertion in L^pro^ have host specificity and affect the virulence of the virus, the properties of two field O/SEA/Mya-98 lineage strains, O/BY/CHA/2010 (without the 70-nt deletion and amino acid insertion) and O/Mya98/JX/2010 (containing the 70-nt deletion and single amino acid insertion in L^pro^), were first investigated and compared. The results indicated that O/BY/CHA/2010 affected both pigs and cattle; however, O/Mya98/JX/2010 affected only pigs and did not cause any clinical manifestations in cattle. Reverse genetics was subsequently used to produce genetically engineered chimeric viruses and define the genetic basis of the host specificity, and it was determined that the 70-nt deletion in the S fragment combined with the leucine insertion in L^pro^ was a genetic determinant of the virulence of O/SEA/Mya-98 FMDV that resulted in attenuation of the virus in bovines.

## RESULTS

### A 70-nt deletion in the S fragment within the 5′ UTR and a leucine/valine insertion in L^pro^ coexisted in several swine origin O/SEA/Mya-98 FMDV strains.

**FIG 1:**
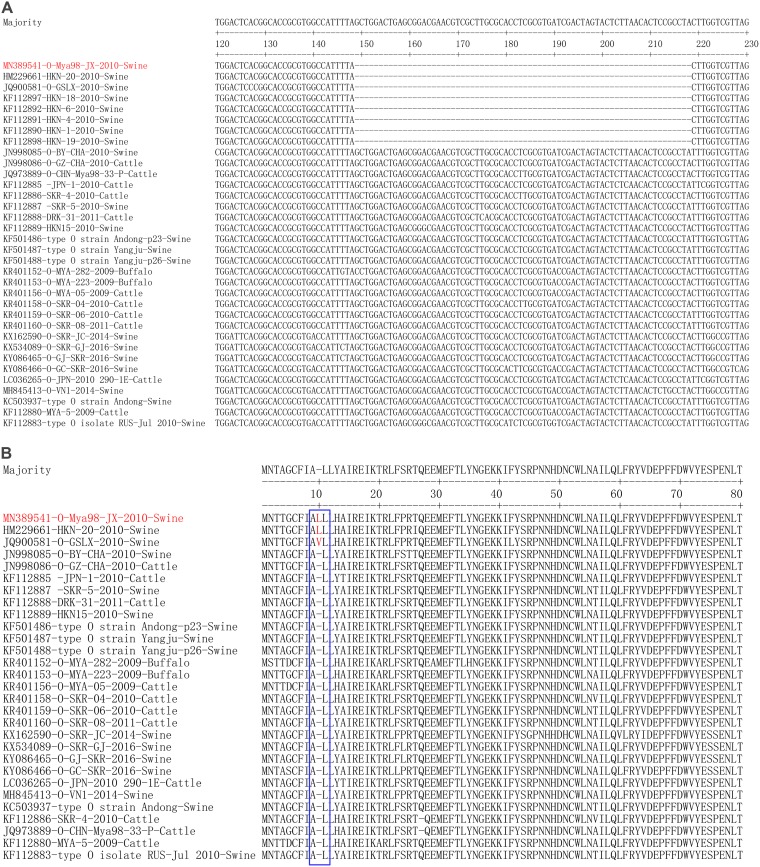
A 70-nt deletion in the S fragment within the 5′ UTR and an amino acid insertion in L^pro^ occurred naturally in several O/SEA/Mya-98 FMDV strains. (A) Alignment of the 5´ UTR sequences of O/SEA/Mya-98 lineage FMDV strains. A 70-nt deletion in the S fragment within the 5´ UTR was identified in O/Mya98/JX/2010, HKN/20/2010, O/GSLX/2010, HKN/18/2010, HKN/6/2010, HKN/4/2010, HKN/1/2010, and HKN/19/2010 after comparison with other O/SEA/Mya-98 FMDV strains. (B) Alignment of the O/SEA/Mya-98 lineage FMDV polyprotein amino acid sequences available in GenBank. A leucine/valine insertion at position 10 of L^pro^ which coexisted with the 70-nt deletion in the S fragment was identified in O/Mya98/JX/2010, HKN/20/2010, and O/GSLX/2010 strains.

Our laboratory previously isolated an O/SEA/Mya-98 FMDV strain, O/Mya98/JX/2010 (GenBank accession number MN389541), from swine that included a 70-nt deletion in the S fragment of the 5´ UTR of the viral genome. To further investigate the genomic characteristic of O/Mya98/JX/2010, the complete genome sequences of O/SEA/Mya-98 lineage FMDVs available in GenBank were collected and analyzed. A comparison of the complete genome sequences revealed that two other viral strains, HKN/20/2010 (GenBank accession number HM229661) and O/GSLX/2010 (GenBank accession number JQ900581), also included similar deletions within the S fragment at nt 148 to 217 (Fig. 1A). We also analyzed all the 5′ UTR sequences of O/SEA/Mya-98 lineage FMDV strains available in GenBank, which showed that another five FMDV strains isolated in Hong Kong, China, in 2010 also included this deletion (Fig. 1A). This indicated that the 70-nt deletion naturally occurred in the 5´ UTR of several O/SEA/Mya-98 lineage FMDV strains. The polyprotein sequences of these strains (with complete genome sequences available in GenBank) were further compared and analyzed. Interestingly, we found that the strains that included the 70-nt deletion also contained a 3-nt insertion in the L gene. The amino acid sequence alignment of L^pro^ indicated that the 3-nt insertion encoded a leucine or valine at position 10 of L^pro^, and O/Mya98/JX/2010 was similar to HKN/20/2010, which contained a leucine insertion within L^pro^ (Fig. 1B).

### Strain O/Mya98/JX/2010 caused clinical signs in pigs but not in cattle.

A 10-amino-acid deletion in 3A protein has been identified as an altered virulence and host tropism of serotype O Cathay topotype FMDV (3), and previous studies also suggest that there are some other viral determinants that might be associated with the virulence and host range specificity of serotype O FMDV (23, 24). In the present study, the O/SEA/Mya-98 lineage FMDV strains with the 70-nt deletion in the S fragment of 5´ UTR were all isolated from pigs, which implied that these strains possibly had a restricted host range that affected only pigs. To compare and evaluate the susceptible host species and virulence of the viruses with and without the observed mutations, two O/SEA/Mya-98 viruses, O/Mya98/JX/2010 (including the 70-nt deletion within the 5´ UTR and a leucine insertion in L^pro^) and O/BY/CHA/2010 (without the nucleotide deletion within the 5´ UTR or amino acid insertion in L^pro^; GenBank accession number JN998085), were used to carry out further studies.

**FIG 2:**
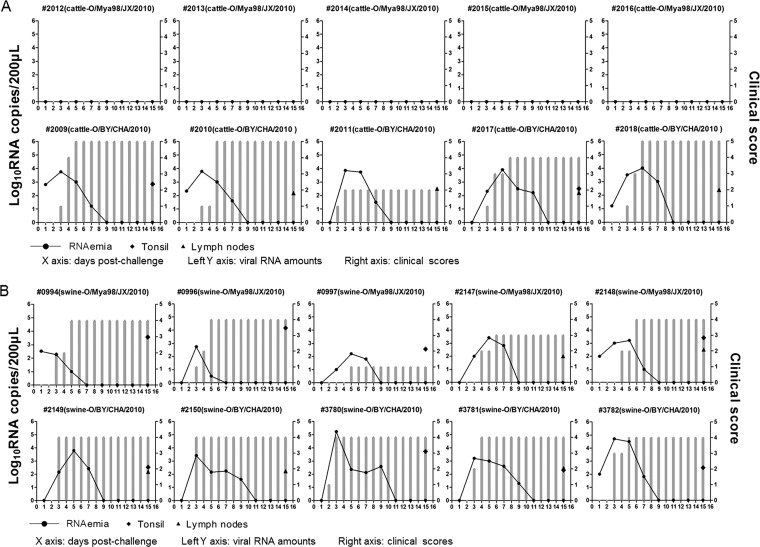
Strain O/Mya98/JX/2010 was not pathogenic in cattle. (A) Ten cattle were challenged by intradermal inoculation with 10^7^ TCID_50_/animal of O/Mya98/JX/2010 or O/BY/CHA/2010. The clinical signs were monitored daily, and viral RNA levels in the blood were measured at 1, 3, 5, 7, 9, 11, 13, and 15 days postchallenge (dpc). The viral RNAs in the bovine submandibular lymph nodes and tonsil tissues were measured using qPCR at 15 dpc. RNAemia is defined as the detection of FMDV RNA in blood samples of challenged animals. The *x* axes show the day postchallenge. The left *y* axes represent the viral RNA amounts in the blood and tissues; the right *y* axes represent the clinical scores at different days postchallenge (gray bars). (B) Ten pigs were inoculated intramuscularly with 10^7^ TCID_50_ of O/Mya98/JX/2010 or O/BY/CHA/2010. The clinical signs and viral RNAs in the blood and organ tissues accessible by the virus were determined as described above to evaluate the infection status of the viruses in pigs.

Either FMDV at 10^7^ tissue culture infective dose (TCID_50_)/animal was used to inoculate the animals. A high dose of FMDV could cause a clear clinical outcome of FMD in both cattle and pigs (25, 26). Five cattle and five pigs were challenged with O/Mya98/JX/2010, and another five cattle and five pigs were challenged with O/BY/CHA/2010. All of the challenged cattle and pigs were monitored daily for clinical signs of disease for 2 weeks (Fig. 2). The clinical scores were recorded to describe the clinical signs of disease, as previously described (27). Briefly, one or more lesions per foot is recorded as 1 point, and a mouth, nostril, or tongue lesion beyond the inoculation site is recorded as 1 point. The maximum score per animal is 5. It was observed that the five cattle challenged with O/Mya98/JX/2010 showed no signs of clinical disease. In contrast, the five cattle challenged with O/BY/CHA/2010 revealed significant clinical signs of FMD (foot lesions) (Fig. 2A). All pigs challenged with O/Mya98/JX/2010 or O/BY/CHA/2010 developed typical clinical manifestations of FMD; however, O/Mya98/JX/2010 induced slightly impaired or delayed clinical signs relative to O/BY/CHA/2010. O/Mya98/JX/2010 caused clinical scores of 0 to 2 at 4 days postinfection (dpi), and O/BY/CHA/2010 caused clinical scores of 3 to 4 at 4 dpi in the challenged pigs (Fig. 2B).

The viral RNAs in the blood and several organ tissues accessible by the virus (submandibular lymph nodes and tonsil tissues) were also detected to evaluate the infection status of the viruses in the challenged animals. Viral RNA was undetectable in the blood and organ tissues from the five cattle challenged with O/Mya98/JX/2010, and we could not recover O/Mya98/JX/2010 from these cattle, whereas viral RNA was detectable in the blood and organ tissues from the five cattle challenged with O/BY/CHA/2010 (Fig. 2A), and the virus could also be recovered. As for the pigs, viral RNA was detectable from the viral replication organ tissues and blood of all pigs after viral challenge. However, the pigs challenged with O/Mya98/JX/2010 revealed a shorter viral RNA duration phase (Fig. 2B). Viral RNA existed in the blood of O/Mya98/JX/2010-challenged pigs from 1 to 7 dpi, and it could not be detected after 7 to 9 dpi. In the pigs challenged with O/BY/CHA/2010, viral RNA disappeared after 9 to 11 dpi.

FMDV-specific antibody titers and antibody against nonstructural protein (NSP) 3ABC were further detected. As shown in Table 1, all animals inoculated with O/BY/CHA/2010 developed significant levels of FMDV-specific antibodies starting at 5 to 9 dpi. However, the cattle challenged by O/Mya98/JX/2010 did not develop significant levels of FMDV-specific antibodies. All animals revealed detectable NSP 3ABC antibodies except for the cattle challenged by O/Mya98/JX/2010. These results suggest that O/Mya98/JX/2010 affected only pigs, not cattle.

**TABLE 1:** NSP serology detected by 3ABC kit and specific antibody titers measured by LPBE assay for O/Mya98/JX/2010 and O/BY/CHA/2010 FMDV strains

Virus	Antibody	Species	No.	Antibody titer at dpi:							Species	No.	Antibody titer at dpi:						
				1	5	7	9	11	13	15			1	5	7	9	11	13	15
O/Mya98/JX/2010	3ABC	Cattle	2012	−	−	−	−	−	−	−	Pig	0994	−	−	−	−	+	+	+
			2013	−	−	−	−	−	−	−		0996	−	−	−	−	+	+	+
			2014	−	−	−	−	−	−	−		0997	−	−	−	−	−	+	+
			2015	−	−	−	−	−	−	−		2147	−	−	**−**	−	−	**+**	**+**
			2016	−	−	−	−	−	−	−		2148	−	−	**−**	−	**+**	**+**	**+**
	Specific antibody	Cattle	2012	1:11	1:22	1:45	1:45	1:45	1:45	1:45	Pig	0994	<1:8	1:360	1:360	1:360	1:360	1:360	1:180
			2013	<1:8	1:11	1:11	1:45	1:45	1:45	1:45		0996	<1:8	1:180	1:720	1:720	1:720	1:720	1:360
			2014	<1:8	<1:8	<1:8	1:45	1:45	1:45	1:45		0997	<1:8	1:16	1:45	1:180	1:720	1:1,024	1:1,024
			2015	<1:8	<1:8	1:11	1:22	1:45	1:45	1:45		2147	1:11	1:45	1:720	1:720	1:720	>1:1,024	>1:1,024
			2016	<1:8	<1:8	<1:8	1:22	1:45	1:45	1:45		2148	<1:8	1:90	1:360	1:720	1:1,024	>1:1,024	>1:1,024
O/BY/CHA/2010	3ABC	Cattle	2009	−	−	**−**	**−**	**+**	**+**	**+**	Pig	2149	−	−	+	+	**+**	**+**	+
			2010	−	−	**−**	**+**	**+**	**+**	**+**		2150	−	−	**−**	**+**	**+**	**+**	**+**
			2011	−	−	**−**	**−**	**+**	**+**	**+**		3780	−	−	**−**	**+**	**+**	**+**	**+**
			2017	−	−	**−**	**−**	**+**	**+**	**+**		3781	−	−	**−**	**+**	**+**	**+**	**+**
			2018	−	−	**−**	**−**	**+**	**+**	**+**		3782	−	−	**−**	**+**	**+**	**+**	**+**
	Specific antibody	Cattle	2009	<1:8	>1:1,024	>1:1,024	>1:1,024	>1:1,024	>1:1,024	>1:1,024	Pig	2149	<1:8	1:22	1:720	>1:1,024	>1:1,024	>1:1,024	>1:1,024
			2010	<1:8	>1:1,024	>1:1,024	>1:1,024	>1:1,024	>1:1,024	>1:1,024		2150	<1:8	<1:8	1:22	1:180	1:360	1:360	1:360
			2011	1:11	1:180	1:720	>1:1,024	>1:1,024	>1:1,024	>1:1,024		3780	1:11	1:360	>1:1,024	>1:1,024	>1:1,024	>1:1,024	>1:1,024
			2017	<1:8	1:180	1:720	>1:1,024	>1:1,024	>1:1,024	>1:1,024		3781	1:11	1:90	1:360	1:720	>1:1,024	>1:1,024	>1:1,024
			2018	1:11	1:360	>1:1,024	>1:1,024	>1:1,024	>1:1,024	>1:1,024		3782	<1:8	1:180	1:720	1:1,024	>1:1,024	>1:1,024	>1:1,024

### Construction of recombinant viruses with or without mutations in the S fragment and L^pro^.

**FIG 3:**
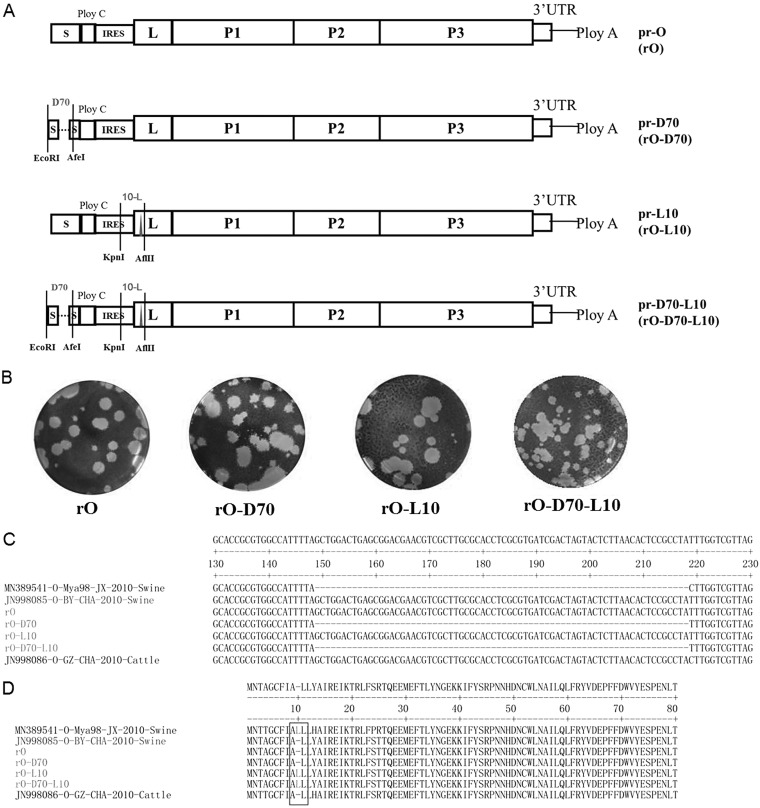
Construction and rescue of four recombinant viruses. (A) Schematic representation showing the constructs of prO, pr-D70, pr-L10, and pr-D70-L10. (B) Plaque assays of rO, rO-D70, rO-L10, and rO-D70-L10 on BHK-21 cells. (C) Alignment of the 5′-UTR region sequences of the four recombinant viruses, O/Mya98/JX/2010, and O/BY/CHA/2010, as well as another O/SEA/Mya-98 lineage FMDV strain, O/GZ/CHA/2010. (D) Alignment of the polyprotein sequences of the four recombinant viruses and O/Mya98/JX/2010, O/BY/CHA/2010, and O/GZ/CHA/2010. A solid black rectangle indicates the leucine insertion in L^pro^.

To investigate whether the 70-nt deletion in the S fragment and the leucine insertion in L^pro^ affect the host range of serotype O FMDV, strain O/BY/CHA/2010, which presented the ability to affect both cattle and pigs, was used for further study. Four recombinant viruses, strains rO-D70 (with the 70-nt deletion in S fragment), rO-L10 (with the leucine insertion in L^pro^), rO-D70-L10 (with both the 70-nt deletion in S fragment and the leucine insertion in L^pro^), and rO (without the 70-nt deletion or the leucine insertion), were rescued based on O/BY/CHA/2010 by using the reverse-genetics system. The characteristics of the four rescued viruses are shown in Fig. 3. We constructed plasmid prO and three derivatives of plasmid prO (pr-D70, pr-L10, and pr-D70-L10) containin a 70-nt deletion in the S fragment, a leucine insertion in L^pro^, or a 70-nt deletion combined with the leucine insertion in L^pro^. Transfection of prO plasmid in BHK-21 cells generated a wild-type O/BY/CHA/2010 FMDV with a full-length S fragment region and a wild-type L^pro^ (rO), while transfection of pr-D70 generated a mutant O/BY/CHA/2010 strain with a 70-nt deletion in the S fragment region (rO-D70), transfection of pr-L10 generated a mutant O/BY/CHA/2010 strain with a leucine insertion at position 10 of L^pro^ (rO-L10), and transfection of pr-D70-L10 generated a mutant O/BY/CHA/2010 strain with a 70-nt deletion in the S fragment region and a leucine insertion at position 10 of L^pro^ (rO-D70-L10) (Fig. 3A). An indirect immunofluorescence assay (IFA) was carried out using the polyclonal antibodies specific for FMDV to identify the rescued viruses in the BHK-21 cells, and clear green fluorescence was observed in the cells infected by the rescued viruses (data not shown). The plaque-forming assay revealed that all four rescued viruses caused significant visible cytopathic effect (CPE) in BHK-21 cells (Fig. 3B). These data suggest that the four viruses had been successfully rescued. The sequences of the S fragment and L gene of the four rescued viruses were determined and analyzed, which confirmed the successful introduction of the designed deletion or insertion in the viruses (Fig. 3C and D). To further confirm that apart from the 70-nt deletion in the S fragment and a leucine insertion in L^pro^, the other regions of the four viruses were completely identical and the introduced mutations were stable in the viral genome, the viral genome sequences of the fourth-passage progeny viruses in BHK-21 cells were determined and compared. The results showed that the progeny viruses stably hold the designed mutations. Apart from the designed deletion or/and insertion, some synonymous substitutions were found in the polyprotein coding sequence. However, no amino acid mutation, deletion, or insertion was observed, which suggested that there was no difference among the viral proteins and noncoding regions of the four viruses except for the designed deletion or/and insertion.

### The 70-nt deletion in the S fragment and the leucine insertion in L^pro^ decreased viral replication of O/BY/CHA/2010 in bovine-derived BTY cells.

**FIG 4:**
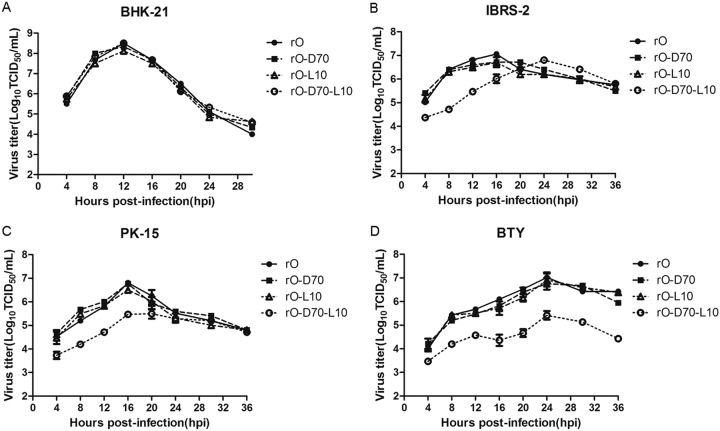
rO-D70-L10 showed significantly decreased infective ability for bovine cells compared to that of rO. One-step growth curves are shown for the four recombinant viruses in BHK-21 (A), IBRS-2 (B), PK-15 (C), and BTY (D) cells. The cells were infected with each virus at a multiplicity of infection (MOI) of 0.5 and maintained at 37°C. Samples of supernatants were harvested at the indicated times, and viral titers were measured.

The coexistence of a 70-nt deletion in the S fragment and a leucine insertion in L^pro^ was observed in O/Mya98/JX/2010 in comparison to O/BY/CHA/2010. To investigate the role of the 70-nt deletion in the S fragment and the single leucine insertion in L^pro^ in the pathogenicity of FMDV, the four rescued viruses described above were used to evaluate their replication ability in different cell lines *in vitro*. The growth characteristics of the four viruses in BHK-21 cells, swine-derived PK-15 and IBRS-2 cells, and bovine-derived BTY cells were determined. One-step growth curves showed that there was no remarkable difference in the viral titers among the four viruses in BHK-21 cells (Fig. 4A). In PK-15 and IBRS-2 cells, the dual-mutation virus rO-D70-L10 revealed slower growth ability than that of the other three viruses (Fig. 4B and C). However, in BTY cells, rO-D70-L10 showed significant replicative disadvantages over rO, rO-D70, and rO-L10 and caused a clearly decreased cytopathogenic effect (Fig. 4D). These results indicate that the 70-nt deletion in the S fragment combined with the leucine insertion in L^pro^ contributed to the restricted growth of the virus on bovine cells.

### The 70-nt deletion in the S fragment together with the leucine insertion in L^pro^ contributed to decreased pathogenicity of O/BY/CHA/2010 in cattle.

**FIG 5:**
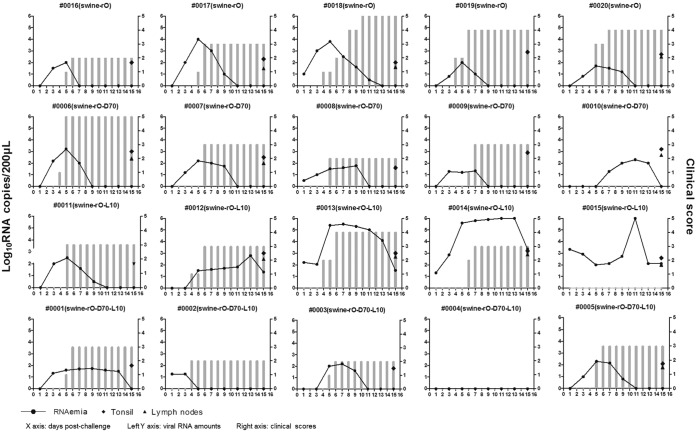
rO-D70-L10 showed slightly reduced pathogenicity in pigs compared with rO. Four groups of pigs, with five pigs in each group, were inoculated intramuscularly with 10^7^ TCID_50_ of rO, rO-D70, rO-L10, and rO-D70-L10, respectively. Clinical signs were monitored daily, and viral RNA levels in the blood were measured at 1, 3, 5, 7, 9, 11, 13, and 15 dpc. The viral RNAs in the submandibular lymph nodes and tonsil tissues were measured using qPCR at 15 dpc. RNAemia is defined as the detection of FMDV RNA in blood samples of challenged animals. The *x* axes show the days postchallenge. The left *y* axes represent the viral RNA amounts in the blood and tissues, and the right *y* axes represent the clinical scores at different days postchallenge (gray bars).

**FIG 6:**
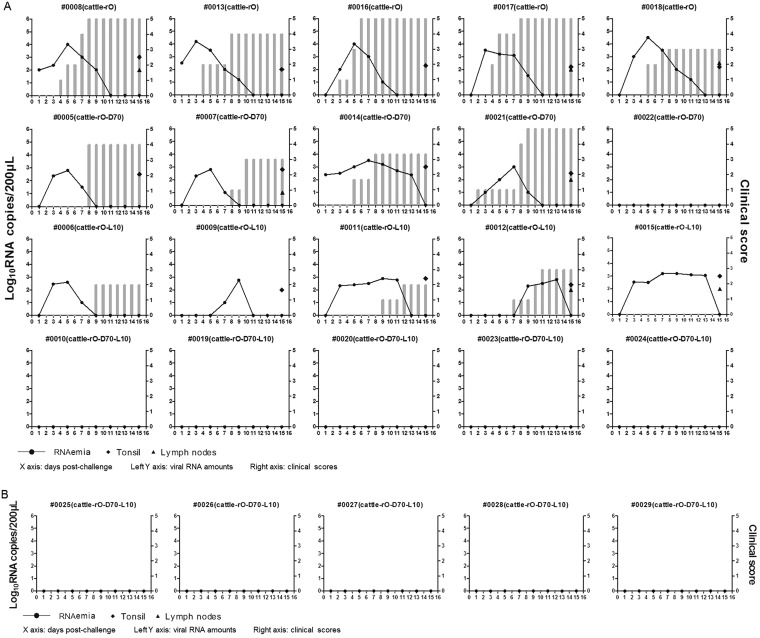
rO-D70-L10 did not cause clinical manifestations in cattle. (A) Twenty cattle were challenged by intradermal inoculation with 10^7^ TCID_50_/animal of rO, rO-D70, rO-L10, or rO-D70-L10. The clinical signs were monitored daily, and viral RNA levels were measured at 1, 3, 5, 7, 9, 11, 13, and 15 dpc. The viral RNAs in the submandibular lymph nodes and tonsil tissues were measured using qPCR at 15 dpc. (B) Five cattle were challenged by intradermal inoculation with 10^8^ TCID_50_/animal of rO-D70-L10. Clinical signs and viral RNAs in the blood and submandibular lymph nodes and tonsil tissues were determined as described above. RNAemia is defined as the detection of FMDV RNA in blood samples of challenged animals. The *x* axes show the days postchallenge. The left *y* axes represent the viral RNA amounts in the blood and tissues; and the right *y* axes represent the clinical scores at different days postchallenge (gray bars).

To investigate whether the 70-nt deletion in the S fragment and the leucine insertion in L^pro^ contributed to bovine attenuation of rO *in vivo*, the four rescued viruses (rO, rO-D70, rO-L10, and rO-D70-L10) were further used for animal challenge experiments. Four groups of animals, with each group including five cattle and five pigs, were inoculated with 10^7^ TCID_50_/animal of different viruses. Meanwhile, an additional group of five cattle was challenged with a 10-fold-higher dose of rO-D70-L10 (10^8^ TCID_50_/cattle) to confirm its virulence in cattle. RNAemia is defined as the detection of FMDV RNA in blood samples of challenged animals. All pigs challenged with rO, rO-D70, or rO-L10 manifested with RNAemia. rO-D70, rO-L10, and rO-D70-L10 showed decreased pathogenicity in pigs compared to rO, and rO-D70-L10 showed the lowest pathogenicity. One of the pigs (no. 0004) challenged by rO-D70-L10 showed an absence of RNAemia and clinical signs of disease (Fig. 5). However, in cattle, rO-D70-L10 did not cause visible clinical signs of disease or RNAemia in any of the challenged animals (Fig. 6A), even at a high dose of inoculum (10^8^ TCID_50_/cattle) (Fig. 6B). Besides, rO-D70 and rO-L10 caused significantly impaired clinical signs of disease and RNAemia in cattle in comparison to rO (Fig. 6A). This suggested an additive effect of the 70-nt deletion in the S fragment with the single leucine insertion in L^pro^ to decrease the virulence of rO.

FMDV-specific antibody and NSP 3ABC antibodies were further measured to confirm the infectious status of the animals challenged by various viruses. The results showed that cattle challenged with rO-D70-L10 did not develop significant levels of FMDV-specific antibodies, and no detectable NSP 3ABC antibodies were observed (Table 2). The five cattle inoculated with a high dose of rO-D70-L10 (10^8^ TCID_50_/cattle) also showed undetectable NSP 3ABC antibodies and extremely low levels of FMDV-specific antibodies (Table 3). Both NSP 3ABC and FMDV-specific antibodies were detected in all of the challenged pigs except one (no. 0004), which when challenged by rO-D70-L10 showed an absence of NSP 3ABC antibodies (Table 2). All of these data confirmed that there was a synergistic and additive effect of the 70-nt deletion within the S fragment and a single leucine insertion in L^pro^ on the virulence of serotype O FMDV in cattle. Deletion of the 70 nt from the 5′ end and insertion of one leucine in L^pro^ in the O/SEA/Mya-98 FMDV resulted in a critical attenuation of the virus in bovines.

**TABLE 2:** NSP serology detected by 3ABC kit and specific antibody titers measured by LPBE assay for FMDV recombinant viruses

Virus	Antibody	Species	No.	Antibody titer at dpi:							Species	No.	Antibody titer at dpi:						
				1	5	7	9	11	13	15			1	5	7	9	11	13	15
rO	3ABC	Cattle	0008	−	−	−	−	+	+	+	Pig	0016	−	−	−	−	−	±	+
			0013	−	−	−	−	+	+	+		0017	−	−	−	−	−	−	+
			0016	−	−	−	−	−	±	+		0018	−	−	−	−	+	+	+
			0017	−	−	−	−	+	+	+		0018	−	−	−	−	+	+	+
			0018	−	−	−	−	+	+	+		0020	−	−	−	−	−	+	+
	Specific antibody	Cattle	0008	＜1:8	1:90	1:720	>1:1,024	>1:1,024	>1:1,024	>1:1,024	Pig	0016	＜1:8	1:45	1:360	1:360	1:720	1:720	1:720
			0013	1:11	1:90	1:1,024	>1:1,024	>1:1,024	>1:1,024	>1:1,024		0017	＜1:8	1:180	1:720	1:720	>1:1,024	>1:1,024	>1:1,024
			0016	＜1:8	1:180	1:720	1:1,024	>1:1,024	>1:1,024	>1:1,024		0018	1:11	1:360	1:720	>1:1,024	>1:1,024	>1:1,024	>1:1,024
			0017	1:8	1:90	1:1,024	>1:1,024	>1:1,024	>1:1,024	>1:1,024		0018	＜1:8	1:720	1:1,024	>1:1,024	>1:1,024	>1:1,024	>1:1,024
			0018	1:11	1:360	1:720	1:720	>1:1,024	>1:1,024	>1:1,024		0020	＜1:8	1:360	1:720	1:720	>1:1,024	>1:1,024	>1:1,024
																		
rO-D70	3ABC	Cattle	0005	−	−	−	−	+	+	+	Pig	0006	−	−	−	−	±	+	+
			0007	−	−	−	±	+	+	+		0007	−	−	−	−	−	−	+
			0014	−	−	−	+	+	+	+		0008	−	−	−	−	−	−	+
			0021	−	−	−	+	+	+	+		0009	−	−	−	−	−	+	+
			0022	−	−	−	−	−	−	−		0010	−	−	−	−	−	−	−
	Specific antibody	Cattle	0005	1:11	1:45	1:720	>1:1,024	>1:1,024	>1:1,024	>1:1,024	Pig	0006	＜1:8	1:45	1:360	1:720	1:720	1:720	>1:1,024
			0007	＜1:8	1:11	1:22	1:90	1:720	1:720	1:720		0007	＜1:8	1:45	1:180	1:360	1:720	1:720	1:1,024
			0014	1:11	1:360	1:720	>1:1,024	>1:1,024	>1:1,024	>1:1,024		0008	1:11	1:45	1:90	1:360	1:360	1:720	1:720
			0021	＜1:8	1:180	>1:1,024	>1:1,024	>1:1,024	>1:1,024	>1:1,024		0009	1:11	1:22	1:360	1:720	>1:1,024	>1:1,024	>1:1,024
			0022	＜1:8	1:22	1:90	1:180	1:180	1:180	1:180		0010	＜1:8	1:45	1:45	1:45	1:90	1:180	1:180
																		
rO-L10	3ABC	Cattle	0006	−	−	−	−	−	−	+	Pig	0011	−	−	−	−	−	−	+
			0009	−	−	−	−	+	+	+		0012	−	−	−	−	−	−	+
			0011	−	−	−	−	−	−	+		0013	−	−	−	−	−	+	+
			0012	−	−	−	+	+	+	+		0014	−	−	−	−	−	+	+
			0015	−	−	−	−	+	+	+		0015	−	−	−	−	−	−	−
	Specific antibody		0006	1:11	1:22	1:180	1:180	1:180	1:180	1:180	Pig	0011	＜1:8	1:180	1:360	1:360	1:1,024	>1:1,024	>1:1,024
			0009	1:11	1:45	1:45	1:45	1:720	1:720	1:360		0012	＜1:8	1:90	1:360	1:360	1:720	1:720	1:1,024
			0011	＜1:8	1:11	1:45	1:180	1:180	1:180	1:180		0013	＜1:8	1:90	1:360	1:720	>1:1,024	>1:1,024	>1:1,024
			0012	1:22	1:45	1:180	1:720	>1:1,024	>1:1,024	>1:1,024		0014	1:11	1:45	1:360	>1:1,024	>1:1,024	>1:1,024	>1:1,024
			0015	＜1:8	1:11	1:45	1:90	1:180	1:180	1:180		0015	＜1:8	1:11	1:22	1:45	1:45	1:45	1:45
																			
rO-D70-L10	3ABC	Cattle	0010	−	−	−	−	−	−	−	Pig	0001	−	−	−	−	−	+	+
			0019	−	−	−	−	−	−	−		0002	−	−	−	−	−	−	+
			0020	−	−	−	−	−	−	−		0003	−	−	−	−	−	−	+
			0023	−	−	−	−	−	−	−		0004	−	−	−	−	−	−	−
			0024	−	−	−	−	−	−	−		0005	−	−	−	−	−	−	+
	Specific antibody	Cattle	0010	＜1:8	＜1:8	＜1:8	1:45	1:45	1:45	1:45	Pig	0001	1:11	1:360	1:720	1:720	>1:1,024	>1:1,024	>1:1,024
			0019	＜1:8	＜1:8	1:11	1:11	1:11	1:11	1:22		0002	1:22	1:720	1:720	1:1,024	1:1,024	>1:1,024	>1:1,024
			0020	＜1:8	＜1:8	1:22	1:22	1:22	1:22	1:22		0003	＜1:8	1:90	1:360	1:360	1:1,024	1:1,024	1:1,024
			0023	＜1:8	＜1:8	1:11	1:22	1:22	1:22	1:22		0004	1:22	1:45	1:45	1:45	1:45	1:45	1:45
			0024	＜1:8	＜1:8	＜1:8	1:11	1:11	1:11	1:22		0005	＜1:8	1:180	1:360	1:720	1:720	>1:1,024	>1:1,024

**TABLE 3:** NSP serology detected by 3ABC kit and specific antibody titers measured by LPBE assay in cattle challenged with high dose of rO-D70-L10

Antibody	Species	No.	Antibody titer at dpi:						
			1	5	7	9	11	13	15
3ABC	Cattle	0025	−	−	−	−	−	−	−
		0026	−	−	−	−	−	−	−
		0027	−	−	−	−	−	−	−
		0028	−	−	−	−	−	−	−
		0029	−	−	−	−	−	−	−
Specific antibody	Cattle	0025	＜1:8	＜1:8	＜1:8	1:11	1:22	1:22	1:22
		0026	＜1:8	＜1:8	＜1:8	1:22	1:45	1:45	1:45
		0027	＜1:8	＜1:8	1:11	1:11	1:11	1:11	1:11
		0028	＜1:8	＜1:8	1:11	1:22	1:45	1:45	1:45
		0029	＜1:8	＜1:8	1:11	1:11	1:22	1:22	1:22

## DISCUSSION

Compared to other picornaviruses, FMDV has a wide host range (28). In different countries or areas, different animals play different roles as the natural epidemiological or maintenance host under various conditions. For example, African buffalo are an important maintenance host in Africa (1). The prevalent virus strains also determine the epidemiological hosts and viral distribution status, because different virus strains may have different epidemiological characteristics and host ranges, such as the Cathay topotype serotype O FMDV, which causes clinical disease only in pigs (4). The evolution and mutations in the viral genome sequences can mediate adaptation of FMDV to different hosts, resulting in changes in host cell specificity and antigenicity (29). For instance, the nonstructural protein 3A is identified as the primary determinant for the restricted host range and a C-terminal truncation has been proved to be responsible for the bovine-attenuated phenotype (3). Therefore, monitoring sequence changes in the viral genome of FMDV is essential for clarifying the pathogenic and virulence changes of the virus.

During 2010-2011, the serotype O Mya-98 lineage FMDV caused a series of high-profile FMD outbreaks in China (21, 30). An unexpected 70-nt deletion within the S fragment of the 5´ UTR was identified in several of the FMDV strains, and the deletion was located at positions 148 to 217 in the 5′ UTR. This deletion resulted in a shorter predicted RNA stem-loop for the S fragment. However, the effect of this deletion on the virulence of FMDV remains unclear. To explore the influence of the 70-nt deletion in the viral genome on the virulence or pathogenicity of FMDV, a thorough analysis of the viral genome sequences of different O/SEA/Mya-98 FMDV strains was first carried out. Sequence alignment revealed that an amino acid insertion in L^pro^ at position 10 occurred concurrently with the 70-nt deletion in the S fragment in these mutant viruses, and interestingly, all of these strains were isolated from pigs. Subsequently, we evaluated and compared the replication abilities and pathogenicities of two different field O/SEA/Mya-98 lineage FMDV strains, O/Mya98/JX/2010 (with the 70-nt deletion and the leucine insertion) and O/BY/CHA/2010 (without the nucleotide deletion or amino acid insertion), both *in vitro* and *in vivo*. We found that O/Mya98/JX/2010 showed significantly restricted growth on bovine cells, and it caused disease only in pigs, not cattle; in contrast, O/BY/CHA/2010 could replicate well both on bovine and swine cells, and it affected both pigs and cattle. Thus, we speculated that the 70-nt deletion in the S fragment, the single leucine insertion in L^pro^, or the combined mutations of both the S fragment and L^pro^ may have resulted in the restricted growth of O/Mya98/JX/2010 on bovine cells and contributed to its failure to infect cattle.

To investigate the role of the 70-nt deletion and a single leucine insertion in FMDV, four recombined serotype O viruses were generated based on strain O/BY/CHA/2010, which had been proven to possess the ability to affect both cattle and pigs. The four viruses, one with a complete S fragment and wild-type L^pro^ (rO), one with the 70-nt deletion in the S fragment region (rO-D70), one with a single leucine insertion at position 10 in L^pro^ (rO-L10), and one with the 70-nt deletion combined with the leucine insertion in L^pro^ (rO-D70-L10), were rescued and further studied. The replication and pathogenic characteristics of these viruses were evaluated *in vitro* and *in vivo*. It was very interesting that neither the deletion of 70 nt in the S fragment nor the single leucine insertion in L^pro^ alone was the complete host range determinant of O/BY/CHA/2010. A single mutation of either of the two factors just moderately decreased the virulence of O/BY/CHA/2010 in cattle. Only the coexistence of the mutations in the S fragment and L^pro^ could result in significantly restricted virus growth of O/BY/CHA/2010 on bovine cells and the failure of the virus to infect cattle. There was a synergistic and additive effect of the 70-nt deletion within the S fragment and the single leucine insertion in L^pro^ on the viral virulence of O/BY/CHA/2010. This indicated that the synergistic effect between the noncoding region and viral proteins was also a factor involved in the determination of host specificity of FMDV. A recent study showed that replacement of the VP1 or 3D gene in the genome of FMDV strain O/JPN/2010 by the corresponding fragment derived from O/JPN/2000 could remarkably alter the virulence of O/JPN/2010 in cattle and suckling mice (31), suggesting that multiple genes are involved in the virulence of FMDV. Whether the VP1 and 3D genes have a synergistic effect may be further studied.

The S fragment is engaged in viral replication regulation, contributing to pathogenesis (15), and different kinds of deletions within the S fragment have been observed in FMDVs of different serotypes (21, 32). However, the effect of these deletions on viral pathogenicity was not determined. A comparison of the predicted secondary structures of the S fragment of O/HKN/20/2010, which included a 70-nt deletion within the S fragment, and of O/HKN/15/2010, which contained a complete S fragment, showed that both of the viruses have a single stem-loop; however, it was 35 bp shorter in the apex of O/HKN/20/2010 than in that of O/HKN/15/2010 (21). Whether this change affects viral RNA stability remains unknown. In this study, this deletion partly decreased the virulence of O/BY/CHA/2010 in both pigs and cattle, which implied that the S fragment is associated with viral pathogenicity, and the nucleotides between positions 148 and 217 of the S fragment are involved in this function.

L^pro^ has two isoforms (termed Lab and Lb) that result from initiation at two different start codons separated by 84 nt (33). L^pro^ is well known as an antagonistic factor to suppress host antiviral responses, and it is significantly associated with viral pathogenicity in host cells or animals (6). The precise loss of the Lb coding sequences of FMDV results in different growth characteristics of the virus in different host cells (34). A putative SAP domain (scaffold-attachment factor A [SAFA] and SAFB, apoptotic chromatin-condensation inducer in the nucleus [ACINUS], and protein inhibitor of activated STAT [PIAS] domain) has been identified in FMDV L^pro^ (35). The mutation of two residues at positions 55 and 58 within the SAP domain of L^pro^ has been determined to result in the viral phenotype change (35, 36). The SAP domain mutant virus reveals a stronger ability to induce the expression of several interferon (IFN)-stimulated genes and chemokines than wild-type FMDV, and this mutant virus cannot cause disease in the challenged pigs (9). Therefore, this indicates that L^pro^ is significantly responsible for the viral pathogenicity in the host. A leucine insertion in L^pro^ of O/BY/CHA/2010 in this study also decreased the virulence of the virus in both swine and cattle, which confirmed that L^pro^ is associated with the viral pathogenicity. Whether the mentioned insertion of leucine at position 10 of L^pro^ changed the function of L^pro^ remains unclear, and the involved mechanisms should be further studied.

In the present study, the 70-nt deletion in the S fragment or the single leucine insertion in L^pro^ might moderately decrease the virulence of the virus for the challenged animals. The clinical data in Fig. 6A indicated that the mutant virus with the L insertion caused less disease in cattle than the mutant virus with the 70-nt deletion, while both viruses were as virulent in swine (Fig. 5). This suggests that L^pro^ might be a very critical factor that resulted in the altered host tropism of the virus in cattle. However, the coexistence of mutations in the two regions resulted in a significantly decreased growth ability of the virus in bovine cells and contributed to infection failure in cattle, which indicates that the two mutations synergistically caused a significant change in the viral phenotype and decreased the virulence of the virus in cattle. The concurrence of the two mutations in the O/SEA/Mya-98 lineage FMDV resulted in the altered host range of the virus that enabled the pigs to become the main epidemiological host, and the pathogenicity of the virus tended to be milder. Serotype O is the most common serotype in China. The evolution of these FMDVs to affect only pigs is possibly due to the fact that China is a country with a dense pig population. The large numbers of pigs provide better conditions for the propagation and prevalence of these viruses. Besides, the decreased viral pathogenicity may benefit virus maintenance in the host, because the high pathogenicity or quick replication of the virus may leave the host unable to further support viral maintenance or reproduction (37–39). Under these circumstances, altered S fragment and L genes may have a selective advantage. The 70-nt deletion in the S fragment and a leucine insertion in L^pro^ of O/SEA/Mya-98 lineage FMDVs may be beneficial to the existence and prevalence of the virus in pigs. In summary, this study identified a novel genetic determinant of altered virulence of serotype O FMDVs, generating data that will benefit efforts to understand the viral pathogenicity mechanism of FMDV.

## MATERIALS AND METHODS

### Ethics statement.

All animal experiments were approved and conducted according to the requirements of the Gansu Animal Experiments Inspectorate and the Gansu Ethical Review Committee [license no. SYXK(GAN) 2010-003].

### Cells and viruses.

Baby hamster kidney (BHK-21) cells, IBRS-2 and PK-15 porcine kidney cells, and bovine thyroid (BTY) cells were grown at 37°C and maintained in Dulbecco**’**s modified Eagle**’**s medium (DMEM) (Gibco), supplemented with 10% fetal bovine serum (Gibco). All cells were maintained in a humidified incubator containing 5% CO_2_ at 37°C. FMDV strain O/Mya98/JX/2010 was isolated from an infected pig in Ganzhou City, Jiangxi Province, China, in April 2010. Strain O/BY/CHA/2010 (accession no. JN998085) was isolated from an infected pig in the Baiyun District of Guangzhou City, Guangdong Province, China, in March 2010.

### RNA extraction and cDNA synthesis.

Total RNA was extracted from FMDV-infected cells or tissue samples from virus-infected animals by using an RNeasy kit (Qiagen, Hilden, Germany) according to the manufacturer**’**s specifications and used as the template for cDNA synthesis. SuperScript Moloney murine leukemia virus reverse transcriptase (Life Technologies, Carlsbad, CA) was used for the reverse transcription reactions, and random hexamers were used as primers. The mixtures were incubated at 42°C for 1 h to synthesize the first-strand cDNA. The cDNA was then used as the template for PCR.

### RT-qPCR.

One-step real-time quantitative PCR (RT-qPCR) was performed as previously described by Shaw et al. (40). Briefly, a total volume of 25 μl reaction mixture consisting of 20 μl of RT-PCR master mix reagents (containing the primers) and 5 μl of RNA was added to the appropriate number of wells in a 96-well optical reaction plate (Stratagene, La Jolla, CA). The reaction was performed in an Mx4000 sequence detection system (Stratagene) with an optimized thermal cycling condition. The Stratagene MxPro qPCR software was used for the results analysis, and a threshold cycle (*C_T_*) value was assigned to each reaction as described previously (41). Samples with a *C_T_* value of 35 or less were considered positive for FMDV detection (25).

### Construction of the rO, rO-D70, rO-L10, and rO-D70-L10 infectious clones.

The construction strategy for the rO, rO-D70, rO-L10, and rO-D70-L10 infectious clones is shown in Fig. 3. A novel RNA polymerase I- and II-driven plasmid-based reverse-genetics system was developed by our laboratory previously (25). The full-length infectious clone containing the FMDV O/BY/CHA/2010 sequence was generated based on this reverse-genetics system and named prO. The other three mutant viruses were generated based on prO. A DNA fragment, including the partial 5´ UTR of FMDV O/BY/CHA/2010 with the 70-nt deletion as well as two restriction endonuclease enzyme sites, EcoRI and AfeI, was synthesized, and the fragment was placed into the prO as previously described (42) to generate pr-D70. pr-L10 was constructed with a similar strategy with KpnI and AfIII enzymes, and three nucleotides, TTG, were introduced into the L gene. pr-D70-L10 was constructed based on p-rO-D70, and the KpnI and AfIII enzymes were used to introduce the single leucine insertion at the indicated position. All the constructed plasmids were sequenced.

### Viral rescue and identification.

The purified plasmids prO, pr-D70, pr-L10, and pr-D70-L10 were prepared using Qiagen plasmid midi kits (Qiagen, Hilden, Germany) according to the manufacturer’s protocol. The plasmids were transfected into monolayer BHK-21 cells in 10-cm culture plates using Lipofectamine 2000 (Invitrogen, Carlsbad, CA) according to the manufacturer**’**s instructions. After 48 h posttransfection, the supernatants were harvested. The supernatants were frozen and thawed three times and then were centrifuged at 5,000 × *g* for 10 min at 4°C. The supernatants were blind passaged into BHK-21 cells four times. The rescued viruses were collected and identified after four consecutive passages in BHK-21 cells. The immunofluorescence assay, plaque titration assay, and 50% tissue culture infectious dose (TCID_50_) assay were performed to confirm the successful rescue of the viruses. The viral genomes of the obtained rescued viruses were finally sequenced to ensure that no mutations were introduced.

### TCID_50_ assay.

BHK-21 cells were seeded in 96-well cell culture plates at 4 × 10^4^ cells per well. Titrations were made using serial 10-fold dilutions. The confluent monolayer cells were infected with 10^−1^ to 10^−8^ dilutions of the rescued virus. Replicates of 8 wells (1st to 10th columns) in a 96-well plate were used for each virus dilution (100 μl/well). The cell plate was incubated in a humidified incubator containing 5% CO_2_ at 37°C for 1 h. The cells were then washed with phosphate-buffered saline (PBS) three times to remove the unabsorbed viruses. The infected cells were then maintained with DMEM supplemented with 1% fetal bovine serum for 3 days. For each plate, the number of wells at each dilution with (+) or without (−) a cytopathogenic effect was recorded. The 50% endpoint titer of the virus was determined to calculate the 50% tissue culture infectious dose (TCID_50_).

### Experimental infection of various viruses in cattle and pigs.

A liquid-phase blocking ELISA (LPBE) (43) was performed according to the standard method of the OIE to screen candidate pigs and cattle for animal challenge experiments. All healthy animals that tested negative for FMDV antibodies were used in this study. The animals were housed in disease-secure isolation facilities at Lanzhou Veterinary Research Institute. The cattle, 6 to 7 months old, and pigs, 6 weeks old, were housed in separate rooms. The animals used for challenge by field viruses included the following: group 1 (cattle no. 2012, 2013, 2014, 2015 and 2016; pig no. 0994, 0996, 0997, 2147, and 2148) challenged by O/Mya98/JX/2010 and group 2 (cattle no. 2009, 2010, 2011, 2017, and 2018; pig no. 2149, 2150, 3780, 3781, and 3782) challenged by O/BY/CHA/2010. The animals used for challenge by recombinant viruses included the following: group 3 (cattle no. 0008, 0013, 0016, 0017, and 0018; pigs, 0016, 0017, 0018, 0019, and 0020) challenged by rO; group 4 (cattle no. 0005, 0007, 0014, 0021, and 0022; pig no. 0006, 0007, 0008, 0009, and 0010) challenged by rO-D70; group 5 (cattle no. 0006, 0009, 0011, 0012, and 0015; pig no. 0011, 0012, 0013, 0014, and 0015) and group 6 (cattle no. 0010, 0019, 0020, 0023, and 0024; pig no. 0001, 0002, 0003, 0004, and 0005), challenged by rO-L10 and rO-D70-L10, respectively. The cattle from each group were inoculated intradermally at six sites in the tongue, with 10^7^ or 10^8^ TCID_50_ of each virus. The pigs from each group were inoculated intramuscularly with 10^7^ TCID_50_ of each virus. All animals were monitored daily, and clinical signs were recorded by scoring. The clinical scores for the challenged animals were determined as previously described by Rieder et al. (27). The detailed criteria are as follows: score for mouth, nostril, or tongue lesion beyond inoculation site, 1; score for one lesion per foot, 1; and maximum score for the infected animal, 5. The animals were quickly removed to a separate room when they developed clinical disease for the duration of the experiment. The clotted and heparinized blood samples were collected at 1, 3, 5, 7, 9, 11, and 15 days postinoculation. The collected samples were subjected to viral RNA or antibody detection.

### Antibody detection.

The presence of antibodies against FMDV structural protein (SP) and nonstructural protein (NSP) 3ABC in blood samples was determined by LBPE and an NSP 3ABC detection kit. LPBE was performed as described in the manual of diagnostic tests and vaccines for terrestrial animals (43). Antibodies against the nonstructural protein (NSP) 3ABC were detected using the 3ABC indirect enzyme-linked immunosorbent assay; the NSP 3ABC detection kits were prepared by Lanzhou Veterinary Research Institute, and the detailed procedure was performed as previously described (44).

### Statistical analysis.

All results were presented as means with standard errors (SE). The significance was analyzed using GraphPad Prism (version 5.0) software. Statistical significance was defined as a *P* value of less than 0.05.

### Data availability.

The genomic sequence of O/Mya98/JX/2010 was determined and deposited in the NCBI GenBank database (accession no. MN389541).
